# QR-1011 restores defective *ABCA4* splicing caused by multiple severe *ABCA4* variants underlying Stargardt disease

**DOI:** 10.1038/s41598-024-51203-7

**Published:** 2024-01-06

**Authors:** Melita Kaltak, Petra de Bruijn, Willemijn van Leeuwen, Gerard Platenburg, Frans P. M. Cremers, Rob W. J. Collin, Jim Swildens

**Affiliations:** 1 R&D Department, ProQR Therapeutics, Zernikedreef 9, 2333 CK Leiden, The Netherlands; 2 Department of Human Genetics, Radboud University Medical Center, Geert Grooteplein Zuid 10, 6525 GA Nijmegen, The Netherlands

**Keywords:** Gene therapy, Oligo delivery, RNA splicing

## Abstract

Stargardt disease type 1 (STGD1), the most common form of hereditary macular dystrophy, can be caused by biallelic combinations of over 2200 variants in the *ABCA4* gene. This leads to reduced or absent ABCA4 protein activity, resulting in toxic metabolite accumulation in the retina and damage of the retinal pigment epithelium and photoreceptors. Approximately 21% of all *ABCA4* variants that contribute to disease influence *ABCA4* pre-mRNA splicing. This emphasizes the need for therapies to restore disrupted *ABCA4* splicing and halt STGD1 progression. Previously, QR-1011, an antisense oligonucleotide (AON), successfully corrected splicing abnormalities and restored normal ABCA4 protein translation in human retinal organoids carrying the prevalent disease-causing variant c.5461−10T>C in *ABCA4*. Here, we investigated whether QR-1011 could also correct splicing in four less common non-canonical splice site (NCSS) variants flanking *ABCA4* exon 39: c.5461−8T>G, c.5461−6T>C, c.5584+5G>A and c.5584+6T>C. We administered QR-1011 and three other AONs to midigene-transfected cells and demonstrate that QR-1011 had the most pronounced effect on splicing compared to the others. Moreover, QR-1011 significantly increased full-length *ABCA4* transcript levels for c.5461−8T>G and c.5584+6T>C. Splicing restoration could not be achieved in the other two variants, suggesting their more severe effect on splicing. Overall, QR-1011, initially developed for a single *ABCA4* variant, exhibited potent splice correction capabilities for two additional severe NCSS variants nearby. This suggests the possibility of a broader therapeutic impact of QR-1011 extending beyond its original target and highlights the potential for treating a larger population of STGD1 patients affected by multiple severe *ABCA4* variants with a single AON.

## Introduction

Inherited retinal diseases (IRDs) are a group of rare disorders characterized by severe degeneration of the retina, and so far, > 250 genes have been identified to harbor variants causative for IRDs^1^. Among these, the most frequently mutated gene is *ABCA4*; in fact, it was observed that variants in *ABCA4* are responsible for 30% of all IRD cases^2^. This 128 kb-long gene comprises 50 exons which contain the coding sequence for the membrane-associated ATP-binding cassette subfamily A type 4 (ABCA4) protein. Biallelic variants in *ABCA4* cause a reduced or absent activity of the protein, which results in the manifestation of the most common inherited retinal dystrophy recognized as Stargardt disease (STGD1), as well as other *ABCA4*-linked retinopathies^3^.

The completion of the Human Genome Project^4^ and the resulting advances in personalized medicine provided an arsenal of therapeutic approaches for patients with rare diseases. However, as these are often custom-tailored to meet the specific genetic information of the patient, the development of therapies targeting rare and extremely rare variants is hampered by the high costs of traditional drug development compared to the revenue upon eventual commercialization due to the restricted group of patients that could benefit from the treatment. In STGD1, 46% of all detected unique *ABCA4* variants are “private”, meaning that they have been detected in only one allele among all *ABCA4* alleles associated with STGD1^5^. Notably, there have been a few “N = 1” clinical trials, such as the case of milasen, where Kim and colleagues developed and administered a custom-tailored antisense oligonucleotide (AON) to a single individual facing life-threatening seizures due to Batten’s disease^6^. Milasen was used with the purpose of restoring wild-type (WT) splicing in the *MFSD8* gene under an expanded-access investigational protocol for clinical use which was authorized by the Food and Drug Administration (FDA).

A large part of the “private” variants is expected to influence *ABCA4* pre-mRNA splicing. Splicing variants in *ABCA4* constitute 21% of all disease-associated variants (18% of all unique *ABCA4* variants), prompting previous investigations into potential use of splice-modulating AONs as treatment for STGD1, either by exclusion of pseudo-exons or re-inclusion of skipped exons^7–13^. These are intended for local administration by intravitreal injection, thus minimizing a systemic exposure, while a high target specificity is achieved through their complementarity with the target’s pre-mRNA. AONs can be applied in a mutation-independent manner through allele degradation by ribonuclease H or in-frame exon skipping for variant hotspots. These approaches provide a therapeutic strategy for multiple variants simultaneously in autosomal dominant diseases or diseases where the affected protein is constituted by repetitive sequences and thus tolerates truncations that do not compromise its functionality^14,15^. However, these mechanisms of action cannot be applied for treatment of STGD1 due to its recessive inheritance model and the complex structure of ABCA4. An exception is exon 17, whose skipping does not abolish the activity of ABCA4^16^. Typically, exon re-inclusion by AONs is employed to correct the aberrant splicing caused by one single variant. This requires the identification of strong splice silencers to restore WT splicing. Considering that each variant can influence splicing motifs in a different way, using a single AON for exon re-inclusion as a treatment for multiple variants remains challenging.

In the present study, we explored the AON-mediated exon re-inclusion by a single molecule for multiple *ABCA4* variants. Previously, QR-1011 showed its therapeutic potential for c.5461−10T>C, the most common severe *ABCA4* variant, by restoring WT RNA and protein in 3D human retinal organoids^9^. We identified four other non-canonical splice site (NCSS) variants residing near exon 39 that lead to the same frameshift events as the above-mentioned variant: c.5461−8T>G, c.5461−6T>C, c.5584+5G>A and c.5584+6T>C^17–20^. We define NCSS variants as variants located in the vicinity of exon–intron junctions, which are most likely having an effect on splicing. However, they differ from the canonical splice site variants, which are found at the − 2, − 1, + 1 and + 2 intronic positions. As QR-1011 does not carry a mutation-specific design, but rather targets a strong splicing silencer in *ABCA4* intron 39, we applied it to the four NCSS variants to investigate whether QR-1011 is also able to restore WT *ABCA4* splicing. QR-1011 significantly increased the levels of correctly spliced *ABCA4* for c.5461−8T>G and c.5584+6T>C, thus offering a broader application based on the splicing defect rather than the mutation itself. Collectively, these findings highlight the versatility of QR-1011 and thereby may increase the number of treatable STGD1 patients.

## Materials and methods

### Generation of *ABCA4* midigenes

To introduce the *ABCA4* c.5461−8T>G and the c.5461−6T>C variants, the previously described^9^ WT plasmid and two gBlocks (Integrated DNA Technologies, Coralville, IA, USA) spanning 850 bp of *ABCA4* intron 38 to exon 39 (1:94,477,667–94,476,818, GRCh37), one containing the c.5461−8T>G and the other the c.5461−6T>C variant, were digested with *Box*I and *Bsi*WI (ThermoFisher Scientific, Waltham, MA, USA), then combined and transformed into GT115 competent cells (InvivoGen, San Diego, CA, USA). To create the *ABCA4* c.5584+5G>A and the c.5584+6T>C midigenes, three gBlocks were used. Two 1560-bp gBlocks, spanning a region of the *ABCA4* gene between parts of exon 39 and intron 40 (1:94,476,848–94,475,289, GRCh37), contained each one of the two mutations. The third 1474-bp long gBlock contained the *ABCA4* genomic sequence between parts of intron 40 and intron 41 (1:94,475,338–94,473,896, GRCh37), ending with 31 bp of the midigene backbone, overlapped with the other two gBlocks by 50 bp. The third gBlock was combined with each one of the 1560-bp gBlocks in an overlap-extension PCR with Phusion™ High-Fidelity DNA Polymerase (ThermoFisher Scientific, Waltham, MA, USA) according to the manufacturer’s instructions. Afterwards, the amplicons were used in a second PCR reaction with primers binding in exon 39 (5′-CAGGCTGTGACAGATGTCTATG-3′) and the junction of the multiple cloning site and *rhodopsin* intron 4 that are part of the midigene backbone (5′-GGTACCTCTCCCCGGGTC-3′) as described above. The WT midigene and the generated amplicons were digested with *Box*I and *Sal*I (ThermoFisher Scientific, Waltham, MA, USA), ligated and transformed using the same procedure as described above. The sequences of plasmids were validated with Sanger sequencing.

### Antisense oligonucleotide screening in HEK293 cells

HEK293 cells (ATCC, Manassas, VA, USA) were cultured at 37 °C with 5% CO_2_ in Dulbecco’s Modified Eagle Medium (DMEM; Life Technologies, Waltham, MA, USA) supplemented with 10% Fetal Bovine Serum (Biowest, Nuaillé, France). The cells were transfected with *ABCA4* midigenes and, 24 h later, the AONs were delivered by transfection or gymnotically at a 50 nM or 10 µM-dose, respectively, as described previously^9^. The RNA was extracted 48 h or 120 h post-treatment with the RNeasy Plus Mini Kit (QIAGEN, Hilden, Germany) and 150–500 ng was reverse transcribed using the Verso cDNA Synthesis Kit (ThermoFisher Scientific, Waltham, MA, USA) by following the manufacturer’s protocol. The absolute quantification of *ABCA4* transcripts was assessed with isoform-specific digital PCR assays as reported previously^9^. The visualization of transcripts was carried out with RT-PCR and gel electrophoresis as described previously^18^, and the identified bands were validated with Sanger sequencing. The characterization of PCR artefacts, reported in Supplementary Fig. 2, was assessed with the pGEM-T vector system by following the manufacturer’s instructions (Promega, Madison, WI, USA). The content of the vectors was evaluated with Sanger sequencing using the primer binding in *ABCA4* exon 38 (5′-GAGAATAACCGGACGCTGCT-3′).

The mean percentage of detected full-length *ABCA4* isoforms post-treatment was statistically analyzed using GraphPad Prism 9 with ordinary one-way ANOVA test followed by Dunnet’s multiple comparison test. The potential variations in efficiency among AONs across all treatments were assessed through the use of a one-way ANOVA followed by Tukey’s multiple comparison test. P value ≤ 0.05 was considered statistically significant.

## Results

### In silico splice predictive tools predict different outcomes in splicing for five non-canonical splice site variants in *ABCA4*

*ABCA4* c.5461−10T>C p.[Thr1821Aspfs*6,Thr1821Valfs*13], the most common severe variant associated with STGD1, causes the deletion of either exon 39, or both exons 39 and 40 in *ABCA4*. Previous splicing analyses identified a similar splicing defect in three additional non-canonical splice site (NCSS) variants in *ABCA4*, i.e. c.5461−8T>G, c.5584+5G>A and c.5584+6T>C^19^. The RNA of midigene-transfected HEK293 cells also displayed the presence of *ABCA4* Δexon39 and *ABCA4* Δexons 39–40^9,21^. Next to these variants, we identified one additional NCSS variant, c.5461−6T>C, which is suspected to lead to similar defects in splicing due to its proximity to the above-mentioned variants. The severity assessment carried out by Cornelis et al.^5^ classified c.5584+5G>A and c.5584+6T>C as variants of high severity, whereas c.5461−8T>G and c.5461−6T>C could not be classified due to limited data from STGD1 probands. In addition, according to the ACMG/AMP classification^22^, variants c.5584+5G>A and c.5584+6T>C were attributed the “Likely pathogenic” and “Pathogenic” classifications, respectively, as opposed to the remaining two NCSS variants that were classified as “Likely benign”. These details, together with the frequencies of these NCSS variants, are reported in Table 1.

**Table 1:** List of non-canonical splice site variants upstream and downstream *ABCA4* exon 39 that are assumed to lead to the same splicing defect in *ABCA4* mRNA.

DNA variant	Protein variant^19^	Severity category^5^	ACMG/AMP classification^22^	Number alleles in biallelic cohort^5^	Frequency alleles in biallelic cohort^5^
c.5461−10T>C	p.[Thr1821Aspfs*6,Thr1821Valfs*13]	Severe	Pathogenic	486	0.0435
c.5461−8T>G	p.(Thr1821Aspfs*6)	N/A	Likely benign	2	0.0002
c.5461−6T>C	p.(=)	N/A	Likely benign	1	0.0001
c.5584+5G>A	p.[Thr1821Aspfs*6,Thr1821Valfs*13]	Severe	Likely pathogenic	1	0.0001
c.5584+6T>C	p.[Thr1821Aspfs*6,Thr1821Valfs*13,Glu1863Leufs*33]	Severe	Pathogenic	4	0.0004

N/A, not assessed due to limited data from screened biallelic STGD1 probands.

We implemented in silico splice predictive tools, such as SpliceAI^23^, a deep-learning tool that previously showed to be an accurate tool for predicting the splicing effect of *ABCA4* deep-intronic and coding variants^24^, and Alamut Visual (www.interactive-biosoftware.com/alamut-visual/), which contains four in silico prediction tools, to estimate the potential effect on splicing of the five NCSS variants.

**Figure 1:**
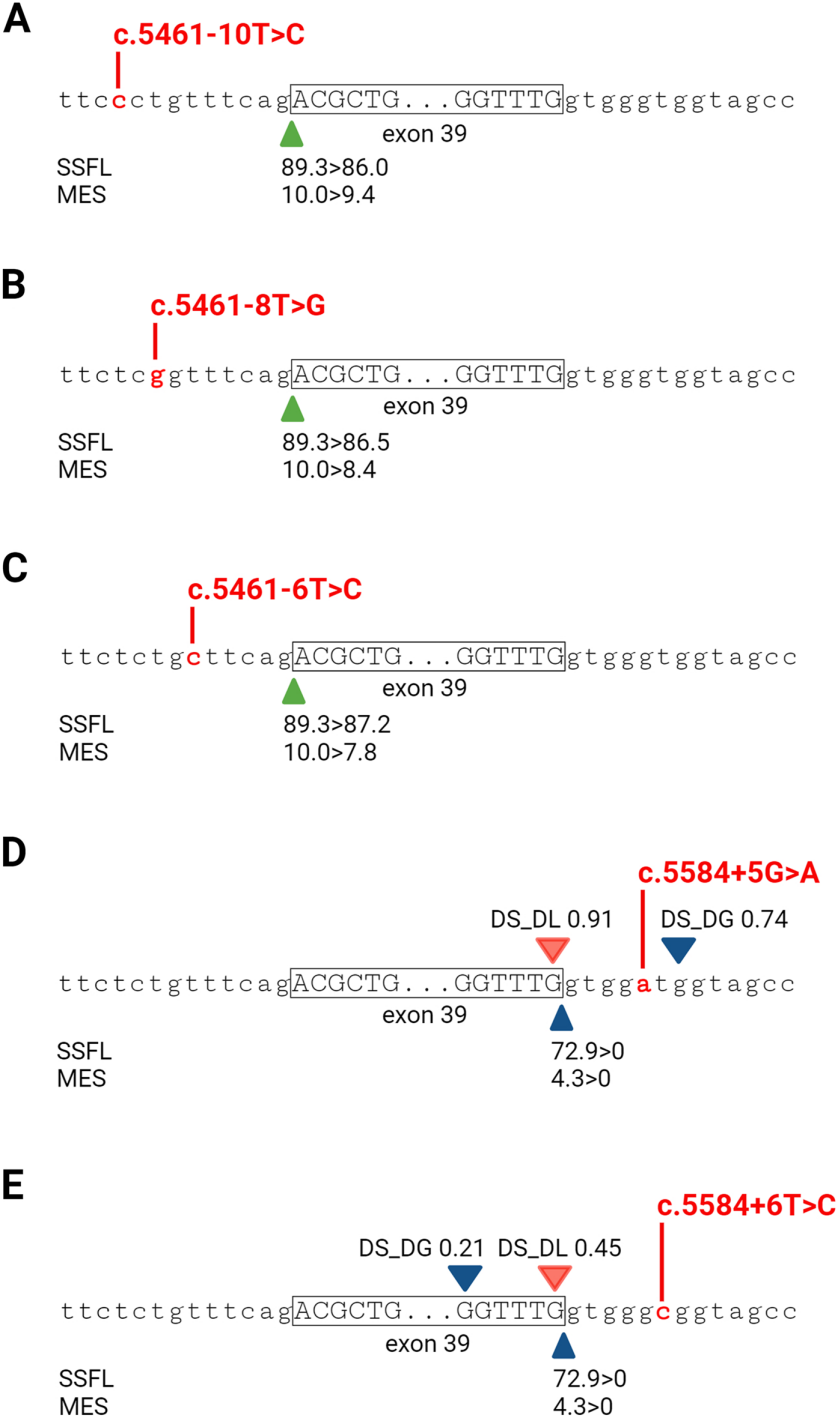
In silico predictions for effects on splicing for *ABCA4* exon 39 non-canonical splice site variants. (**A**) The c.5461−10T>C, (**B**) c.5461−8T>G and (**C**) c.5461−6T>C variants lead to a minor weakening of the downstream splice acceptor site. The presence of (**D**) c.5584+5G>A and (**E**) c.5584+6T>C variants disrupts the upstream splice donor site. These two variants are predicted to activate cryptic splice donor sites 2 nt downstream and 11 nt upstream of their location, respectively. The SpliceAI delta scores (DS) that displayed values > 0.20 threshold are shown on top of the exon 39 sequence, with red and blue triangles depicting the donor loss (DL) and donor gain (DG), respectively. The green and blue triangles below the sequence represent the (predicted) splice acceptor site and splice donor site by SpliceSiteFinder-like (SSFL) and MaxEntScan (MES) tools that are part of the Alamut software.

As shown in Fig. 1, when using the arbitrary threshold for delta score (DS) of > 0.20, SpliceAI predicted that variants c.5584+5G>A and c.5584+6T>C would result in donor gain (DG) and donor loss (DL) effects, with c.5584+5G>A leading to a 7-nt elongation of exon 39 and the c.5584+6T>C variant activating an alternative cryptic splice donor site (SDS) 5 nt upstream of the original one. In contrast, no likely effect on splicing was predicted for the variants c.5461−8T>G and c.5461−6T>C, as well as for the c.5461−10T>C variant, despite previous research confirmed that both c.5461−8T>G and c.5461−10T>C have a detrimental effect on protein translation (Fig. 1A–C).

On the other hand, Alamut predicted a comparable minor weakening of the splice acceptor site (SAS) in exon 39 in the presence of the intron 38 variants, c.5461−10T>C, c.5461−8T>G and c.5461−6T>C (Fig. 1A–C). Variants c.5584+5G>A and c.5584+6T>C are predicted to severely weaken the SDS in exon 39, therefore likely to lead to a loss of recognition of that SDS (Fig. 1D,E). These results suggest that all NCSS variants are likely to affect the recognition of the SAS/SDS of exon 39, and are in line with previous in vitro splicing inquiries for c.5461−10T>C, c.5461−8T>G, c.5584+5G>A and c.5584+6T>C^9,19,21,25^.

### Midigene-based splicing assays confirm the aberrant splicing in non-canonical splice site variants

**Figure 2:**
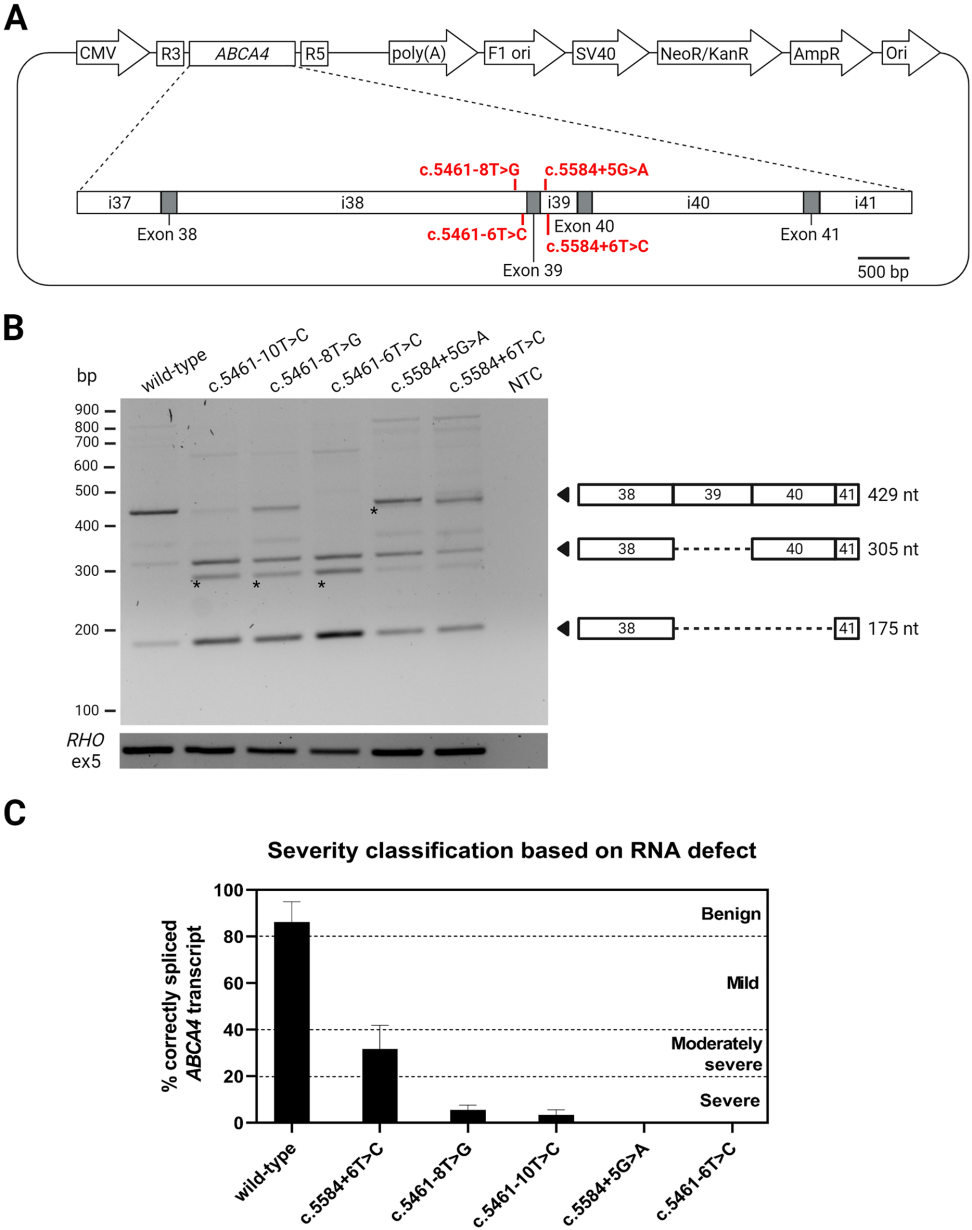
pIC.Rho3-5.ABCA4.exon38-41 midigene to simulate the aberrant splicing. (**A**) The *ABCA4* midigene is spanning 7035 bp of *ABCA4* genomic region between introns 37 and 41 (1:94,480,932—94,473,896, GRCh37) and contains each of the four variants in investigation. (**B**) The expression of pIC.Rho3-5.ABCA4.exon38-41 midigenes containing either the c.5461−10T>C, c.5461−8T>G, c.5461−6T>C, c.5584+5G>A or the c.5584+6T>C variant in HEK293 cells. The WT plasmid served as positive control. NTC, no template control. Asterisks represent PCR artefacts. Uncropped gel images are displayed in Supplementary Fig. 1. Sequences of all products are presented in Supplementary Fig. 2. (**C**) Categorization of variants based on the amount of correctly spliced *ABCA4*. The absolute quantification was carried out using dPCR. Data is shown as mean ± SD, n = 3.

Our hypothesis was that the NCSS variants, due to their localization, could result in aberrant splicing similar to what was previously characterized for the c.5461−10T>C variant. To identify their potential effect on splicing in vitro, the NCSS variants were inserted into an *ABCA4* midigene that contains the genomic region between introns 37 and 41 (as shown in Fig. 2A). The mutant midigenes were expressed in HEK293 cells and their RNA was extracted for analysis. The results confirmed that the NCSS variants have a similar effect on splicing as the c.5461−10T>C variant (Fig. 2B), with all four variants producing out-of-frame isoforms lacking exon 39 or both exons 39 and 40, as seen previously^19,25^. Uncropped gel images are presented in Supplementary Fig. 1. The sequences of identified *ABCA4* transcripts were validated with Sanger sequencing (Supplementary Fig. 2). Based on the expression of correctly spliced *ABCA4* and the severity intervals that derive from unpublished theoretical modelling studies (F.P.M. Cremers) reported in Fig. 2C, c.5461−8T>G, c.5461−6T>C and c.5584+5G>A were labelled as “Severe” since they yielded < 20% of correct transcript. Only the c.5584+6T>C variant produced higher levels of full-length *ABCA4* (31.7 ± 10.2%), which allowed to categorize the variant as “Moderately severe" (Table 2).

**Table 2:** List of non-canonical splice site *ABCA4* variants and their novel effect on RNA and protein, together with the updated ACMG/AMP classification based on the splice defects.

DNA variant	RNA variant	Protein variant	% WT RNA	Severity score based on this splice study	Combined ACMG/AMP classification
c.5461−8T>C	r.[5461_5584del,5461_5714del]	p.[Thr1821Aspfs*6,Thr1821Valfs*13]	5.6	Severe	VUS
c.5461−6T>C	r.[5461_5584del,5461_5714del]	p.[Thr1821Aspfs*6,Thr1821Valfs*13]	0.3	Severe	VUS
c.5584+5G>A	r.[5461_5584del,5461_5714del]	p.[Thr1821Aspfs*6,Thr1821Valfs*13]	0.2	Severe	Likely pathogenic
c.5584+6T>C	r.[5461_5584del,5461_5714del]	p.[Thr1821Aspfs*6,Thr1821Valfs*13]	31.7	Moderately severe	Pathogenic

VUS, variant of unknown significance.

### QR-1011 rescues wild-type *ABCA4* at significant levels in two non-canonical splice site variants

Since all four NCSS variants’ midigenes led to expression of the same isoforms as the c.5461−10T>C variant, we concluded that blocking the previously reported strong hnRNP A1 splice silencer motif in intron 39 might lead to exon re-inclusion^9^. Therefore, we applied the lead AON molecule from those studies, QR-1011, together with three other AON sequences that have different lengths but share the same binding site as QR-1011. All compounds carried a phosphorothioate (PS) backbone with 2’-MOE modifications at all sugar positions. The details of used AONs are reported in Table 3.

**Table 3:** Sequences of antisense oligonucleotides used in the study.

AON	Sequence (5′–3′)	Start position (GRCh37)	End position (GRCh37)
AON32	GGGCCCAUGCUCCAUGGGCCUCGG	94,476,783	94,476,806
QR-1011	AUGCUCCAUGGGCCUCGG	94,476,789	94,476,806
AON59	GCUCCAUGGGCCUCGG	94,476,791	94,476,806
AON60	UGCUCCAUGGGCCUCGG	94,476,790	94,476,806

In order to assess the efficacy of our molecules, we applied a two-step approach. First, the AONs were administered in conjunction with a transfection reagent, which allowed to identify the maximum efficacy of the AONs. Subsequently, we conducted one additional round of experiments using gymnotic uptake treatment to get a better understanding of the splice correction after unaided cellular uptake.

**Figure 3:**
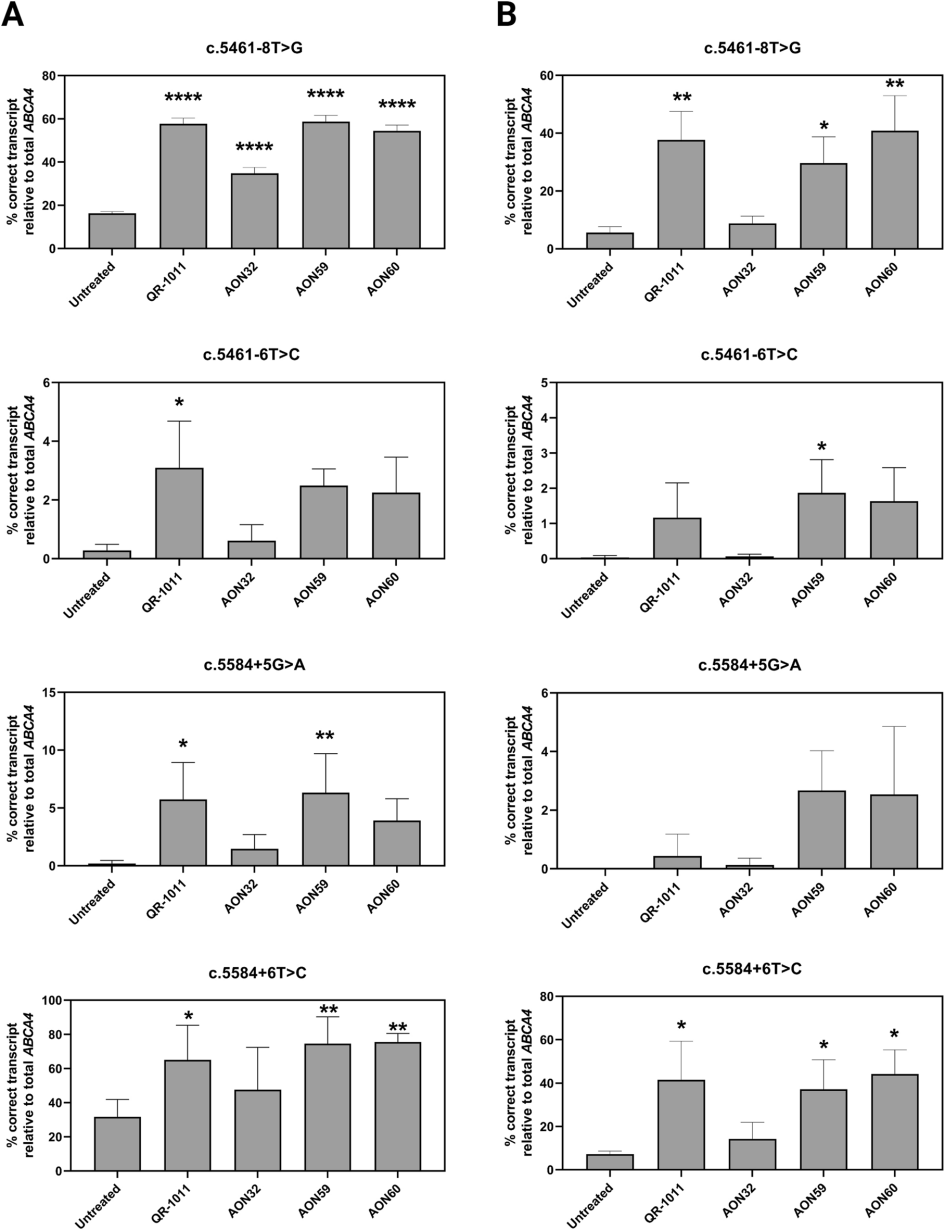
Percentage of correctly spliced *ABCA4* transcript after AON treatment. AONs were delivered by (**A**) transfection (50-nM dose) or (**B**) gymnotic uptake (10-µM dose) to HEK293 cells transfected with c.5461−8T>G, c.5461−6T>C, c.5584+5G>A and c.5584+6T>C midigenes. Data is shown as mean ± SD, n = 3. *p ≤ 0.05, **p ≤ 0.01 and ****p ≤ 0.0001.

Hence, the AONs were administered to midigene-transfected HEK293 cells through transfection, with a 48-h treatment period. Subsequently, the RNA content displayed a significant restoration of *ABCA4* splicing in c.5461−8T>G and c.5584+6T>C, with 58.7 ± 2.9% and 75.5 ± 5.0%, respectively, after treatment with AON59 and AON60 (Fig. 3A). On the other hand, the other two NCSS variants yielded low percentages of rescued exons 39 and 40 following AON treatment. The best-performing molecules for these variants were QR-1011, which achieved 3.1 ± 1.6% of rescue in c.5461−6T>C, and AON59, which led to 6.3 ± 3.4% of rescued *ABCA4* transcript in c.5584+5G>A (Fig. 3A). For variants where QR-1011 did not achieve the highest levels of restoration, it induced levels of splicing restoration that were comparable to the best performing molecule. As expected, AON32 showed the lowest ability for splicing correction in all samples, likely due to its longer length (24 nt) compared to other used molecules. The overview of misspliced *ABCA4* transcripts is displayed in Supplementary Fig. 3A.

Subsequently, this same AON set was administered to midigene-transfected cells by gymnosis. In this case, we observed a decrease in efficiency levels when compared to the transfection treatments, which was in line with our expectations due to the unassisted delivery of the AONs (Fig. 3B). Here, AON60 achieved significant improvements in cells harboring the c.5461−8T>G and c.5584+6T>C variants, with 40.8 ± 7.0% and 44.2 ± 6.5% of correctly spliced *ABCA4*, respectively. QR-1011 performed similarly in the above-mentioned samples, inducing respectively 37.7 ± 5.7% and 41.6 ± 10.3% of WT RNA. The gymnotic treatment in c.5461−6T>C and c.5584+5G>A reflected low percentages of corrected splicing with all used AONs, similarly as in the treatment administered by transfection agent. The percentages of aberrantly spliced *ABCA4* transcripts are shown in Supplementary Fig. 3B.

**Figure 4:**
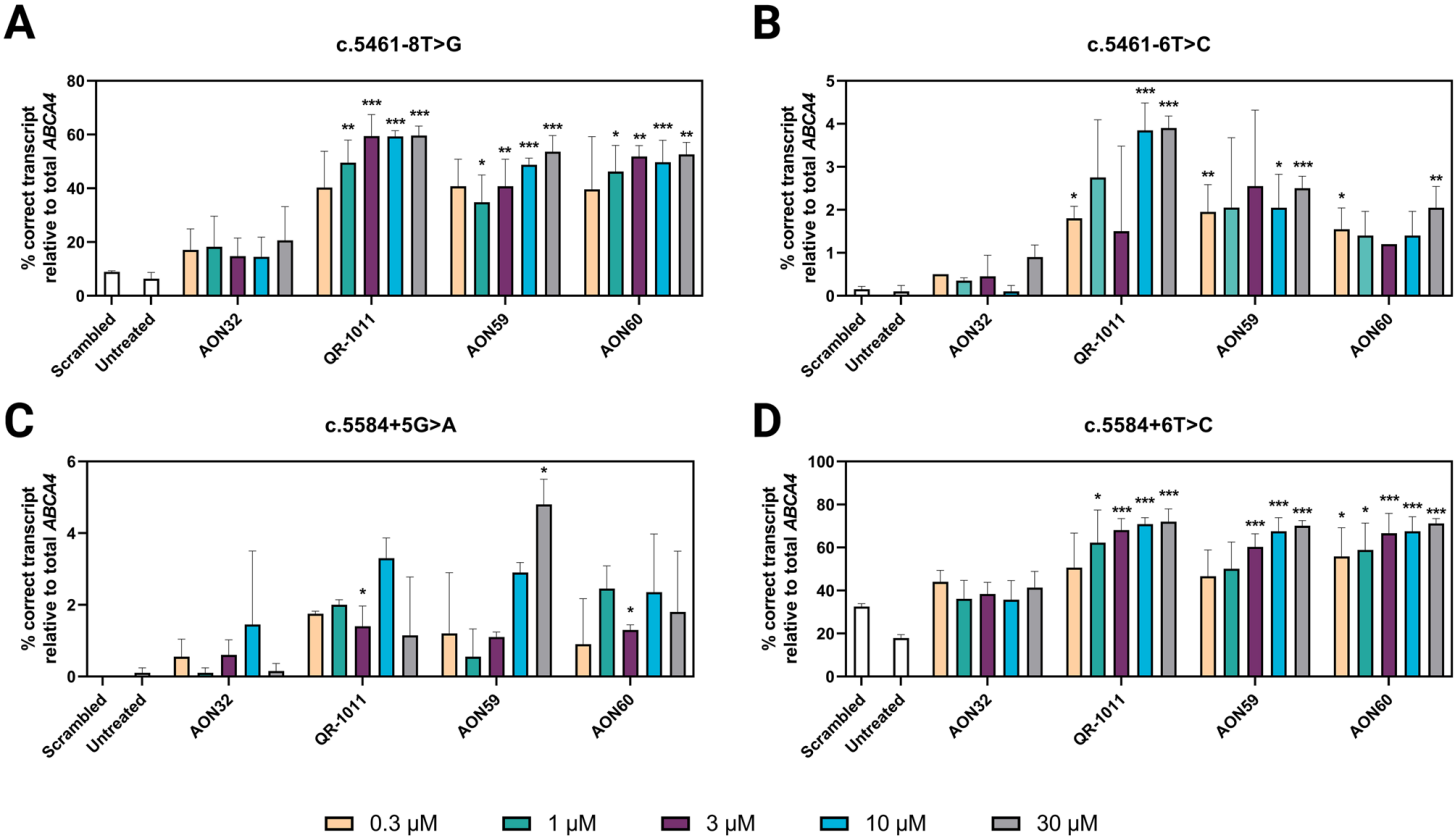
The percentage of correctly spliced *ABCA4* mRNA after AON treatment follows a dose-dependent trend. The midigenes (**A**) c.5461−8T>G, (**B**) c.5461−6T>C, (**C**) c.5584+5G>A and (**D**) c.5584+6T>C were introduced in HEK293 cells and exposed to AONs for 120 h using gymnotic delivery. The results are presented as mean ± SD, n = 3. The statistical significance vs. untreated is indicated by *p ≤ 0.05, ** p ≤ 0.01, ***p ≤ 0.001.

The therapeutic ability of the four AONs showed to behave in a dose-dependent trend in all four variants, as seen previously in experiments on the c.5461−10T>C variant^9^. We observed the maximum effect of all AONs as low as the 3 µM-dose, when seemingly the efficiency reaches a plateau. Out of all AONs and variants tested, only AON59 in c.5584+5G>A (p = 0.0061) showed a significant increase in efficiency at the highest 30-µM dose when compared to the 3-µM dose (Fig. 4). Interestingly, the percentages of rescued WT RNA upon 10-µM dose of all AONs in all samples increased in this experiment, when the treatment was 120 h long, when compared to the 48-h treatment. However, unlike other AONs, only QR-1011 showed a significant increase in its effect after prolonged treatment (Supplementary Fig. 4).

## Discussion

In the present study, we explored the versatility of QR-1011, a previously reported AON initially designed to treat the splicing defect caused by the *ABCA4* variant c.5461−10T>C, by applying it to four additional non-canonical splice site (NCSS) variants in *ABCA4.* These variants all result in deleterious *ABCA4* transcripts lacking either exon 39 or exons 39 and 40 and are associated with STGD1. Our findings demonstrate that QR-1011 has the ability to significantly increase the levels of WT RNA in c.5461−8T>G and c.5584+6T>C, thus expanding its potential as therapeutic strategy for the treatment of STGD1.

Previously, QR-1011 was identified as the most potent splice corrector for the c.5461−10T>C variant, the most frequent severe variant in *ABCA4* associated with STGD1^9^. This AON achieved high levels of restoration of the WT *ABCA4* transcript in 3D human retinal organoids (ROs) homozygous for c.5461−10T>C, which led to significant levels of newly translated ABCA4 protein that co-localized in the outer segments of photoreceptor cells, similar to what was observed in WT ROs^9^. Since QR-1011 doesn’t target the c.5461−10T>C variant, but rather acts by blocking a strong intronic splice silencer (ISS) motif, we hypothesized that it might be beneficial for other *ABCA4* variants causing the same splicing aberration. We identified four NCSS variants located upstream and downstream of exon 39 in *ABCA4* that lead to the same splicing aberrations as the c.5461−10T>C variant.

We employed in silico splice prediction tools, such as SpliceAI and Alamut, to predict the potential effect on splicing that the mentioned NCSS variants have. Interestingly, SpliceAI, which previously showed high reliability in predicting splicing defects imposed by *ABCA4* deep-intronic variants^24^, failed to predict the splicing outcome for the NCSS variants in this study. As reported in Fig. 1, the c.5461−10T>C variant, which served as control, was not assigned a prediction for a splice effect, despite its known effect on the *ABCA4* mRNA splicing process. Therefore, we concluded that the SpliceAI predictions were unreliable for this investigation. As seen previously, SpliceAI fails in collecting the impacts of splice regulatory motifs, such as splice enhancer and silencers, as well as tissue-specific splicing characteristics, and can, consequently, mislead in predicting one variant’s effect on splicing^26,27^. Next to SpliceAI, we consulted Alamut for potential disruption of the splice acceptor site (SAS) and splice donor site (SDS) nearby the NCSS variants. Interestingly, Alamut predicted a slight weakening of the downstream SAS in case of the intron 38 variants and complete obstruction of the SDS in exon 39 in the presence of intron 39 variants (Fig. 1). The assessment of splicing analysis in *ABCA4* midigene constructs confirmed our hypothesis that the four NCSS variants exhibited the same effect on splicing as the c.5461−10T>C variant. Therefore, we applied QR-1011 and three other AONs that target the intronic splice silencer hnRNP A1 in intron 39 with the intention of blocking that silencer motif and induce re-inclusion of exons 39 and 40 in *ABCA4*. Interestingly, when administered through transfection, QR-1011 exhibited significant effect in all four NCSS variants when compared to untreated samples (Fig. 3). We observed significant levels of restoration in c.5461−8T>G and c.5584+6T>C, as opposed to the variants c.5461−6T>C and c.5584+5G>A that displayed < 6% of WT transcript in the transfection treatment and < 2% of WT *ABCA4* in the gymnotic treatment. Based on these results, we attribute the varied sensitivity responses of variants upon the same AON treatment to their more pronounced effect on missplicing, specifically, a greater tendency of the consensus sequence toward the original nucleotide compared to others. Interestingly, the consensus sequence of the human SDS region displays a higher inclination for G than A at the + 5 position (76.7% and 9.1%, respectively), as opposed to the + 6 position where the preference for T was 47.5%. A similar observation was made in the SAS region, where the preference for T at the -8 position slightly drops when compared to its preference at the −6 position^28^. Based on the severity of the effect on splicing and the strong inclination of the consensus sequence toward the original nucleotide, variants c.5461−6T>C and c.5584+5G>A resemble the c.5461−10T>C variant, for which QR-1011 has previously achieved clinically relevant rescue of *ABCA4* pre-mRNA when applied to ROs^9^. This observation suggests that QR-1011 might display higher efficacy in treating the splice defect imposed by the c.5461−6T>C and c.5584+5G>A variants in models with a wider genetic environment, such as ROs.

Since the differences in efficiencies between QR-1011 and the other AONs were not statistically significant and considering the prior selection of QR-1011 as the lead candidate for the c.5461-140T>C variant, we conclude that QR-1011 could be a potential treatment for two additional targets, namely variant c.5461−8T>G and variant c.5584+6T>C, in addition to its original target. These results confirm the flexibility of QR-1011 in treating additional targets beyond its initial intended purpose and expand the number of individuals who could potentially benefit from this splice-modulating therapeutic approach.

Voretigene neparvovec (Luxturna), a gene-augmentation therapy for *RPE65*-associated inherited retinal dystrophy^29^, stands as the only FDA-approved gene therapy targeting an IRD to date. The extensive periods between the discovery and market release jeopardize the progress for development of treatments for rare diseases. In addition, since many IRDs progress slowly, the effects of the drug can only become apparent after significant timeframes, which conflicts with the substantial costs required for sustaining clinical trials, particularly with small patient groups. Despite the appeal of IRDs as attractive targets due to their monogenic nature and the eye being an isolated, hence immune-privileged organ, several phase III clinical trials failed in meeting the FDA’s endpoints due to limited significance in efficacy over the very limited patient groups^30,31^.

A way to tackle the cause of a disease at levels preceding the formation of the mutant protein is to apply personalized therapies that target directly the mutation in the genome or exhibit therapeutic effect at the transcriptional level. Even though these are targeting small numbers of individuals, personalized therapies gain more attention with bigger patient groups, therefore rare and extremely rare variants causal for a disease are being poorly investigated due to unproportional funding needs. In addition, the development and administration of multi-target therapies is obstructed by the vast genetic heterogeneity in individuals. Therefore, it will be difficult to apply a single gene- or transcriptome-editing therapeutic strategy to all individuals suffering from the same disease.

In the current era, the scientific community is adapting methods to expand the target group of personalized approaches. In the present research, we explored whether an AON-based therapy could be implemented to treat several severe variants in *ABCA4* by acting on ISS elements rather than targeting specifically the variants. Suppression of a strong ISS motif with the intent to induce exon re-inclusion has been previously investigated as a therapeutic strategy in *ApoER2*, *GAA* and *SMN2* genes, which underlie Alzheimer disease, Pompe disease and spinal muscular atrophy, respectively^32–34^. While the disease-associated exon skipping in *ApoER2* is not linked to a genetic defect and remains controversial, exon skipping in *GAA* is linked with common genetic variants. On the other hand, AON-based exon re-inclusion in *SMN2* offers benefit to SMA patients that carry different disease-associated variants. The latter became the first AON-mediated approach for exon re-inclusion approved by the FDA, known as nusinersen. Similarly to nusinersen, QR-1011 has demonstrated therapeutic potential that could provide benefit to individuals with various genetic variants underlying STGD1.

Within *ABCA4*, prior inquiries have pinpointed potent single AONs capable of reverting the aberrant splicing associated with multiple disease-associated variants to its WT settings*.* Precisely, Albert and colleagues have reported successful exclusion of a disease-associated pseudo-exon generated upon two neighboring *ABCA4* variants using a single AON^7^. Furthermore, Sangermano et al. have identified an AON candidate with the ability to exclude two distinct disease-associated pseudo-exons that arise in the same genomic region due to two different deep-intronic *ABCA4* variants^11^. However, as far as we are aware, QR-1011 stands as the sole splice-correcting compound able to re-include skipped canonical exons within the field of IRDs. Considering that ABCA4 contains complex domains that are pivotal for its mechanism and conformation, the redirection of splicing can be implemented only to re-include skipped exons or to exclude non-canonical exons. There exists, however, an exception to the rule, identified in exon 17, which was previously observed to encode the simple linker connecting the transmembrane domain 1 (TMD1) and the nucleotide-binding domain 1 (NBD1), and consequently, can be omitted from the coding sequence of *ABCA4*^16^.

In conclusion, we present positive results on the broader applicability of QR-1011 by demonstrating its significant splice-correcting ability for the rare NCSS variants *ABCA4* c.5461−8T>G and c.5584+6T>C. Previous inquiries have determined its restoring ability on the transcriptome and protein levels in gene-edited and patient-derived human ROs homozygous for the frequent c.5461−10T>C variant in *ABCA4*, along with promising safety results from toxicology studies and off-target assessment in other genes^9^. The use of a single molecule capable of treating STGD1 in three different NCSS variants would significantly reduce timelines and costs associated with pre-clinical studies. Moreover, we demonstrate the versatility of AONs by targeting strong splicing motifs instead of specifically focusing on the variant of interest. Therefore, QR-1011 shows to be a potential therapeutic strategy beyond its original target, offering treatment for multiple severe STGD1-associated variants in *ABCA4*.

### Supplementary Information

Supplementary Figures.

## Data Availability

All data generated during this study are available within the paper and its supplementary data. Raw data are available upon request from the corresponding author.
