# Supplementary material for: QR-1011 restores defective ABCA4 splicing caused by multiple severe ABCA4 variants underlying Stargardt disease
Source: Sci Rep. 2024 Jan 6;14:684. doi: 10.1038/s41598-024-51203-7 (PMC10770117; doi:10.1038/s41598-024-51203-7)
Supplement: Supplementary file 1 — Supplementary Figures. [file 41598_2024_51203_MOESM1_ESM.pdf]

SUPPLEMENTARY DATA

QR-1011 restores defective *ABCA4* splicing caused by multiple severe *ABCA4* variants underlying Stargardt disease

Melita Kaltak[1,2], Petra de Bruijn[1], Willemijn van Leeuwen[1], Gerard Platenburg[1], Frans P.M. Cremers[2], Rob W.J. Collin[2] and Jim Swildens[1*]

[1]ProQR Therapeutics, Leiden, the Netherlands

[2]Department of Human Genetics, Radboud University Medical Center, Nijmegen, the Netherlands

ABCA4

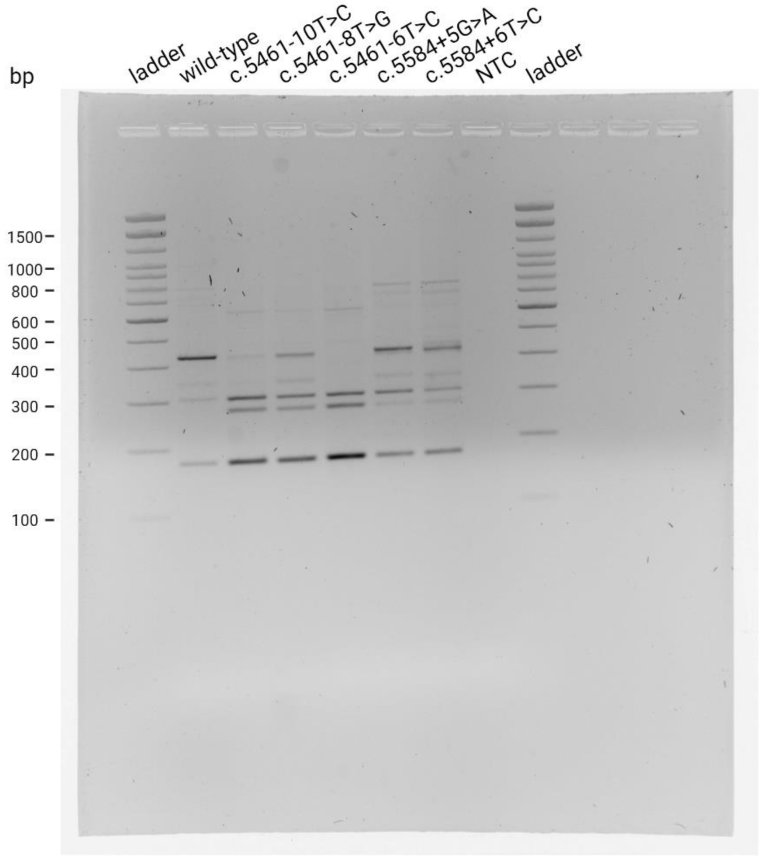

RHO ex5

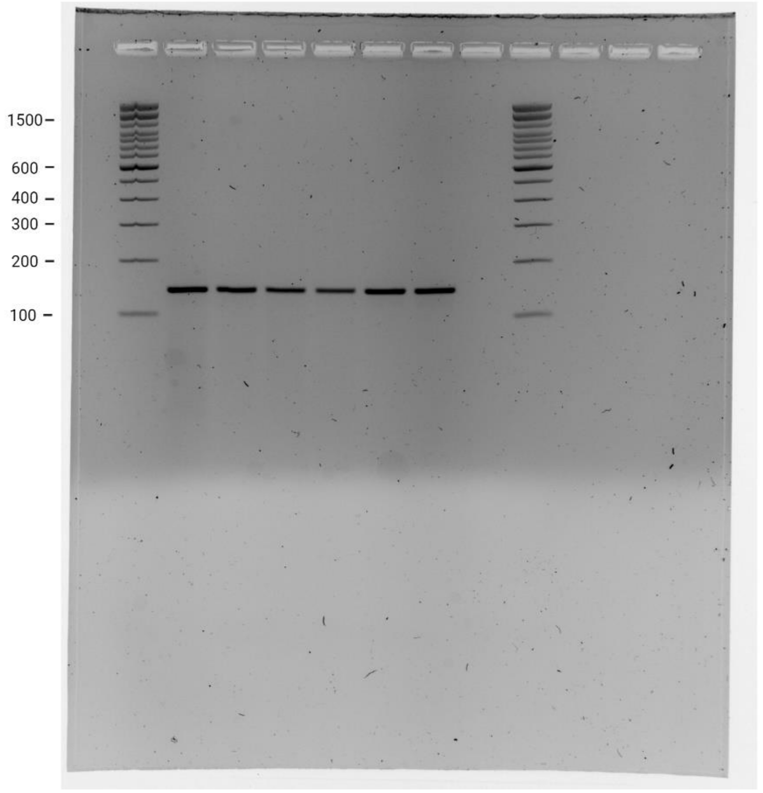

Supplementary Figure 1. Original gels with the RT-PCR from the wild-type, c.5461-10T>C, c.5461-8T>G, c.5461-6T>C, c.5584+5G>A and c.5584+6T>C samples after plasmid expression in HEK293 cells. NTC, no template control.

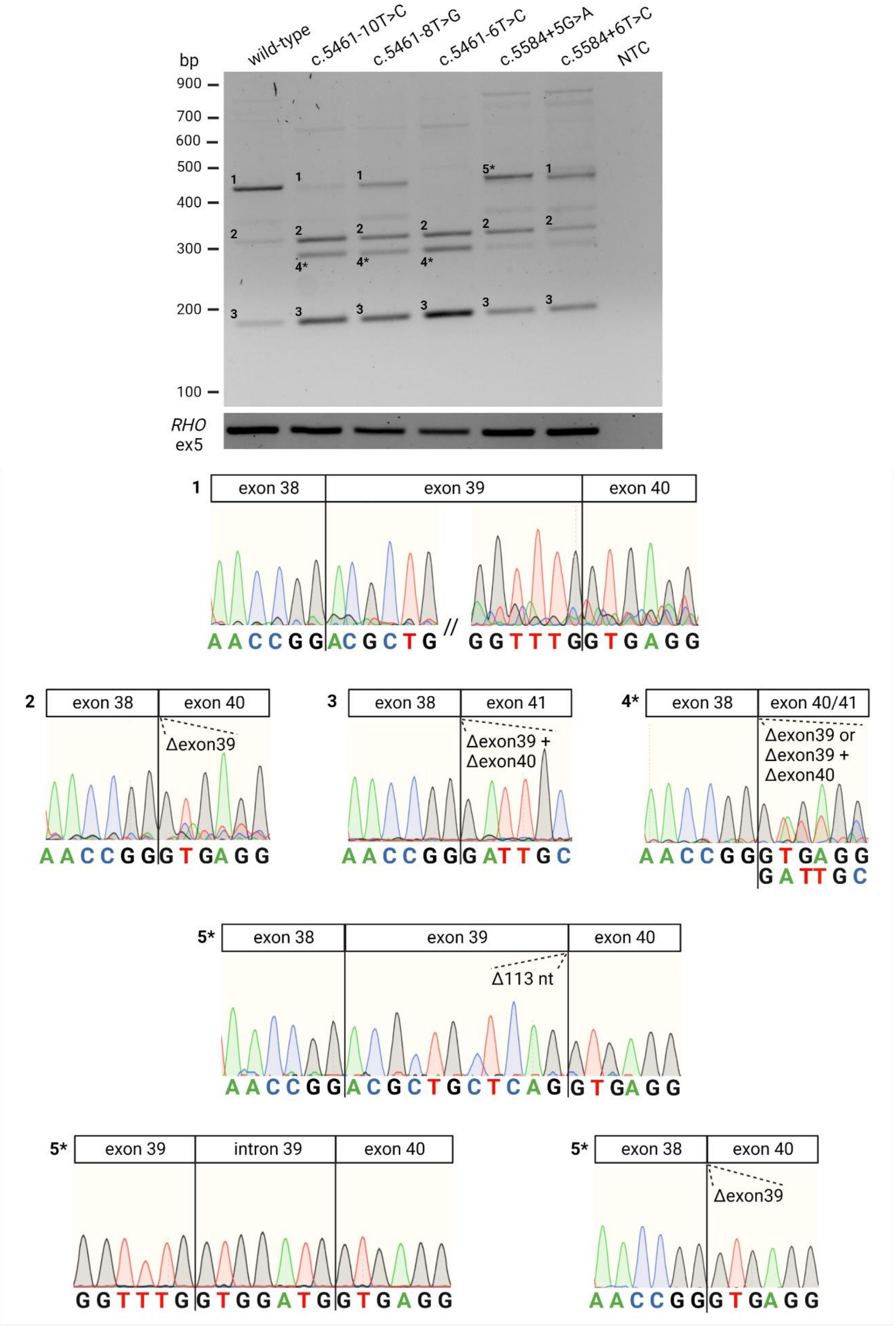

Supplementary Figure 2. RT-PCR of c.5461-10T>C, c.5461-8T>G, c.5461-6T>C, c.5584+5G>A and c.5584+6T>C samples after plasmid expression in HEK293 cells. The WT construct served as

control. NTC, no template control. The sequences of identified bands were validated with Sanger sequencing. Asterisks represent bands containing PCR artefacts.

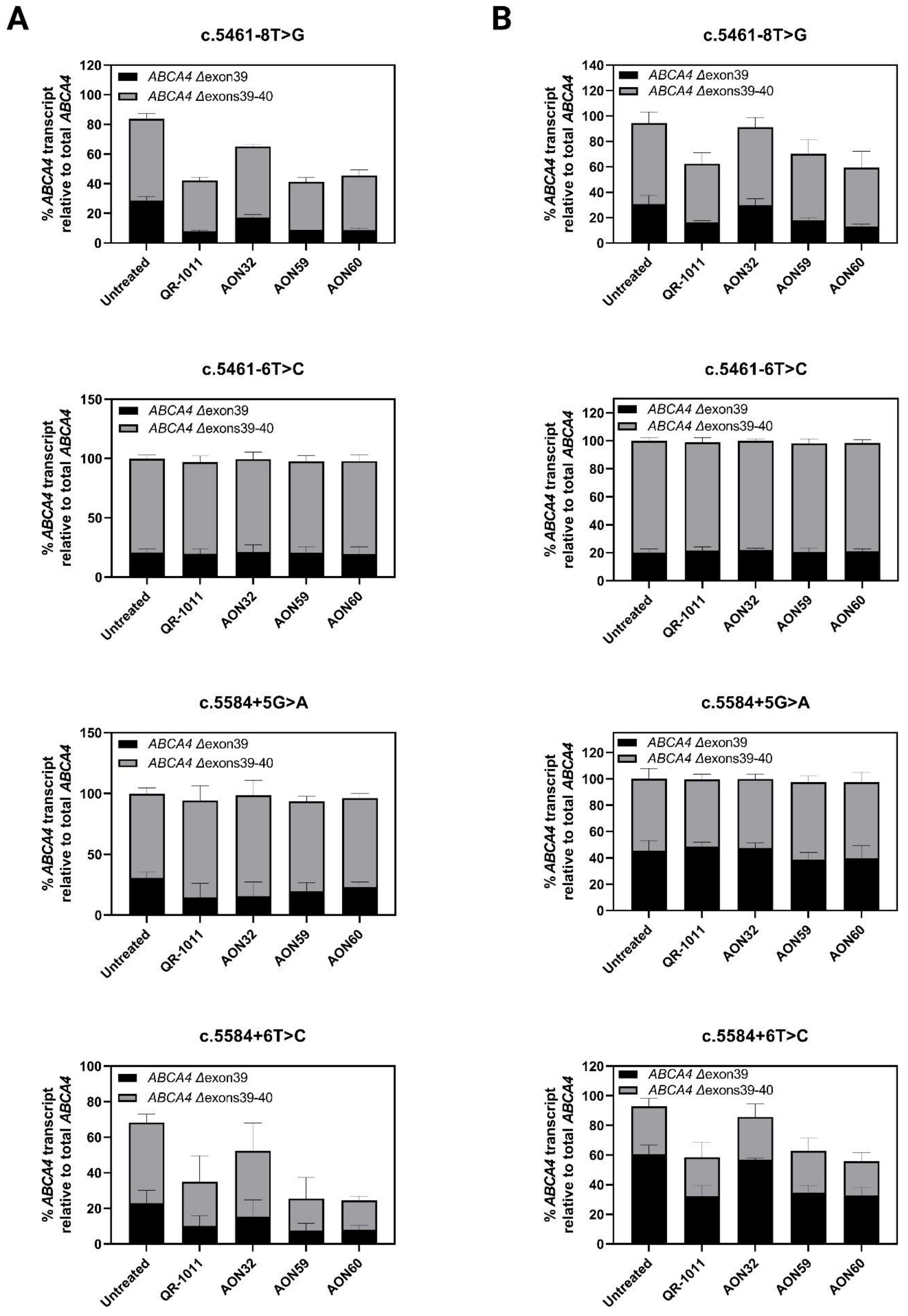

Supplementary Figure 3. Skipping events detected after AON treatment administered by (A) transfection and (B) gymnotic uptake. Data is shown as mean±SD, n=3.

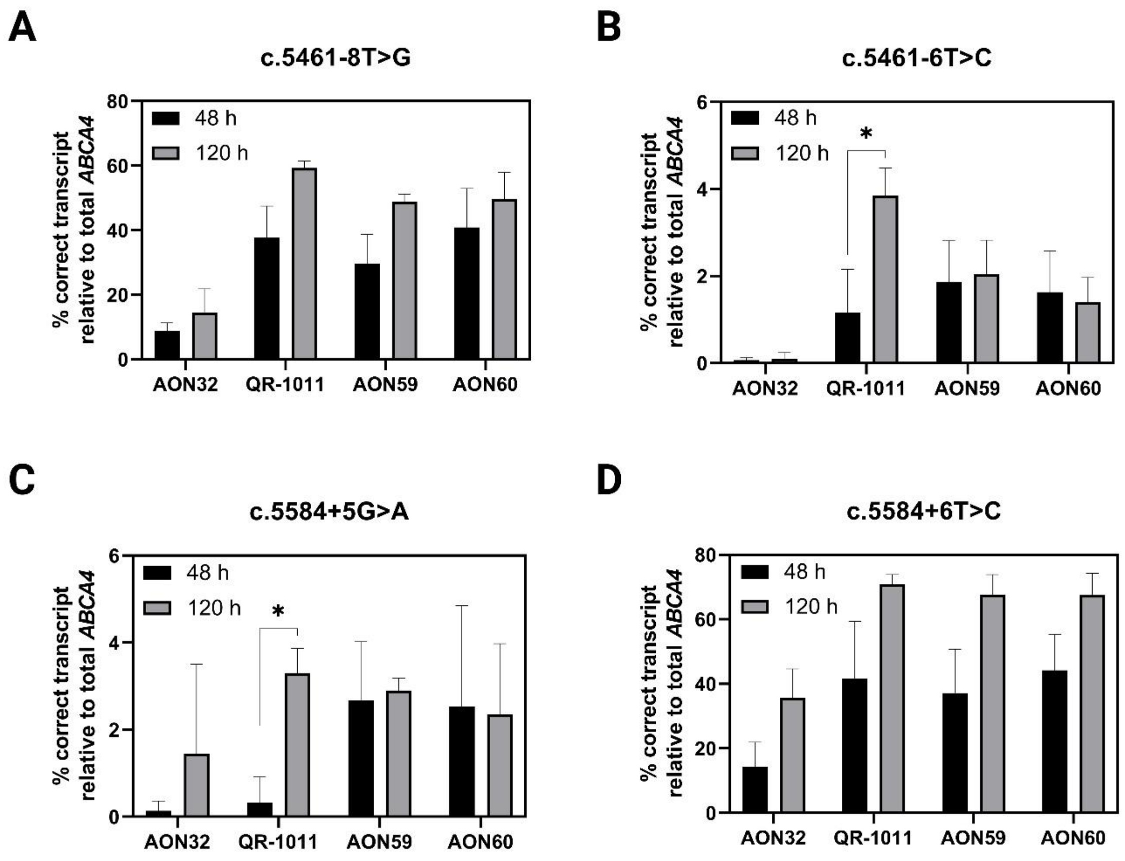

Supplementary Figure 4. Comparison of the AON effect administered by gymnosis after 48 hour-exposure and 120 hour-exposure. Data is shown as mean±SD, n=3, p*≤0.05.
